# Evaluation of resistance to cassava mosaic disease in selected African cassava cultivars using combined molecular and greenhouse grafting tools^☆^

**DOI:** 10.1016/j.pmpp.2018.07.003

**Published:** 2019-01

**Authors:** Jerome Anani Houngue, Martine Zandjanakou-Tachin, Hermine Bille Ngalle, Justin S. Pita, Gilles Habib Todjro Cacaï, Sergine E. Ngatat, Joseph Martin Bell, Corneille Ahanhanzo

**Affiliations:** a Central Laboratory of Plant Biotechnology and Plant Breeding, Department of Genetics and Biotechnology, Faculty of Science and Technique, University of Abomey-Calavi, Benin; b Laboratory of Molecular Plant Pathology, School of Horticulture and Green Space, National University of Agriculture, Porto-Novo, Benin; c Laboratory of Genetics and Plant Breeding, Department of Plant Biology, Faculty of Science, University of Yaoundé I, Yaoundé, Cameroon; d Laboratory of Plant Physiology, Université Félix Houphouët-Boigny, Cote d’Ivoire; e International Institute of Tropical Agriculture (IITA), Nkolbisson, Yaoundé, Cameroon

**Keywords:** *African cassava mosaic virus*, *East African cassava mosaic virus*, Grafting inoculation, CMD resistance gene, *Manihot esculenta*, Resistant cultivars

## Abstract

Cassava mosaic disease (CMD) threatens cassava (*Manihot esculenta*) production in Africa. A total of 24 selected cultivars were screened against CMD using combined molecular and greenhouse grafting tools. Disease severity was recorded for 10 weeks after inoculation and the molecular markers associated with *CMD2* were detected by PCR. CMD severity significantly differed (*P* < 0.0001) among cultivars. Twelve cultivars were morphologically resistant and eight of these possessed *CMD2* and four did not. These results suggest that there are several CMD-resistant cassava cultivars that could be recommended for on-farm production and for conservation and breeding programs.

## Highlights

• Cassava mosaic disease has a major impact on cassava production in sub-Saharan Africa.
• Cassava germplasm were screened for resistance to CMD by grafting tools.
• Eight of the 12 tested cultivars possessed the resistance gene *CMD2*.
• The genetic background of cultivars is the key determinant in plants resisting CMD.
• Resistant cultivars identified in this research could help farmers control CMD damage.

## Table of abbreviations

ACMV: *African cassava mosaic virus*
CMD: Cassava *mosaic* disease
EACMV: *East African cassava mosaic virus*
HR: Highly *resistant* (cultivar)
HS: Highly *susceptible* (cultivar)
QTL: Quantitative trait loci
R: Resistant (*cultivar*)
S: Susceptible (cultivar)
SSR: Simple sequence repeat
SCAR: Sequence-characterized amplified region
WAI: Weeks after inoculation

## 1 Introduction

Cassava (*Manihot esculenta*) is a widely grown root crop and constitutes the most important staple food in tropical and sub-tropical Africa [1]. Several cassava cultivars are grown by farmers. Among these, some are high quality and show flexibility in planting and harvesting times, drought tolerance, ability to adapt to various climatic conditions and good agronomical performance [2,3].

Cassava mosaic disease (CMD) represents one of the main constraints to cassava production. It is caused by 11 cassava mosaic geminivirus species (genus *Begomovirus*, family *Geminiviridae*) in the African continent and the Indian subcontinent of which African cassava mosaic virus (ACMV), African cassava mosaic Burkina Faso virus (ACMBFV), Cassava mosaic Madagascar virus (CMMGV), East African cassava mosaic Cameroon virus (EACMCV), East African cassava mosaic Kenya virus (EACMKV), East African cassava mosaic Malawi virus (EACMMV), East African cassava mosaic virus (EACMV), East African cassava mosaic Zanzibar Virus (EACMZV) and South African cassava mosaic virus (SACMV) have been described in Africa [[4], [5], [6]]. CMD is transmitted by whitefly, *Bemisia tabaci*, and when infected cuttings are reused as planting material [7]. There is a close correlation between the use of infected cuttings and total CMD incidence [8]. Infection rates are greatest in production zones that have high levels of both infected cuttings and whitefly infections [8]. In susceptible cultivars, CMD can cause estimated yield losses of 20–95% [9]. The characteristic symptoms of the disease are leaf mosaic patterns, leaf reduction and leaf chlorosis. However, symptoms can vary among leaves, shoots and plants even within the same cassava variety. Variation in symptoms can be the result of different strains of the same virus or different viruses (species) or both, the sensitivity of the host, plant age and environmental factors, such as soil fertility and soil moisture availability [10]. Control measures against CMD include rogueing (removal of symptomatic plants), the use of virus-free planting materials and the deployment of resistant varieties [11]. The first two options are not only labor intensive and difficult to implement, but also require continuous and long-term interventions. The use of resistant varieties is the most effective solution in mitigating the negative effects of CMD in cassava production [12,13]. This is because this approach reduces yield losses from the disease and reduces the levels of the virus inoculum in the farming system, particularly in varieties that suppress virus accumulation [11].

Several studies have evaluated the resistance of cassava cultivars to CMD [14,15]. However, the infestation pressure is often underestimated or not taken into account because only whitefly transmits the disease during the evaluation period of typical field experiments [16]. Studies have also shown that the rate of CMD transmission by whitefly is relatively low and varies according to the whitefly population at an experimental site [17]. It is therefore necessary to explore other methods, such as molecular and grafting tools, to screen for cassava resistance.

Recently, the mechanical transmission of CMD by grafting was reported to be efficient [18], suggesting this method can be a powerful tool for rapid greenhouse screening of cassava for CMD resistance. To date, three CMD resistance genes, *CMD1* (recessive gene), *CMD2* (major dominant gene) and *CMD3* (quantitative trait loci, QTL, conferring resistance) have been discovered and important molecular markers associated with *CMD2* and *CMD3* have been identified [13,19,20]. The objective of the current study was to identify CMD-resistant cassava cultivars using greenhouse grafting tools and molecular analyses. Our goal is to provide farmers with a recommended list of CMD-resistant cassava cultivars to minimize the damage caused by the disease.

## 2 Materials and methods

### 2.1 Plant material

A total of 216 plants of 24 cassava cultivars from Nigeria, Cameroon and Benin, including 13 local and 11 improved cassava cultivars preferred by farmers, were used in this study. Details of the cassava cultivars are provided in Table 1.

**Table 1:** The 24 cassava cultivars used in this study.

Cultivar name	Type	Origin
8034	Improved	IRAD-Cameroon
92B/0057	Improved	IITA-Benin
Adjatidaho	Local	Benin
Agblehoundo	Local	Benin
Agric-rouge	Local	Benin
Atinwewe	Local	Benin
Ayene Abong-Mbong	Local	Cameroon
BEN/86052	Improved	INRAB-Benin
Excel	Improved	IRAD-Cameroon
Hombete	Local	Benin
Idilerou	Local	Benin
Koleahonme	Local	Benin
Ntollo	Local	Cameroon
Oboul doux	Local	Cameroon
Ouemenou	Local	Benin
Petit manioc	Local	Cameroon
Sanigali	Local	Cameroon
TME 419	Improved	IITA-Cameroon
TME 693	Improved	IITA-Cameroon
TME7	Improved	IITA-Ibadan (Nigeria)
TMS 01/0098	Improved	IITA-Cameroon
TMS 01/1086-55	Improved	IITA-Cameroon
TMS 92/0057	Improved	IITA-Cameroon
TMS 92/0326	Improved	IITA-Benin

IITA, International Institute of Tropical Agriculture.

INRAB, Institut National des Recherches Agricoles du Benin.

IRAD, Institute of Agricultural Research for Development.

### 2.2 Source of inoculum

The source of inoculum was plants of the highly CMV-susceptible cultivar “manioc de table” with disease severity score “5”, exhibiting total distortion on 4/5 of their leaves found to be positive for ACMV via PCR-pretest using JPS1/JSP2 and negative for EACMV by using JSP1/JSP3 primers.

### 2.3 Experimental design

Cuttings of 25–30 cm were submerged in a hot water bath (45–50 °C) for 30 min before potting [21] and found to be free from ACMV and EACMV by PCR indexing two weeks after potting. Nine grafts, sourced from three biological replicate plants of each cassava cultivar, were grafted with six axillary buds (containing inoculum) at the age of three months [17]. The resistant cultivars TME7 and TMS01/0098 were used as controls and underwent the same treatments. The grafting was carried out according to the protocol of Rwegasira et al. [17].

### 2.4 CMD severity in cassava cultivars

The 24 cultivars were evaluated for three months in a controlled environment. Plants of each cultivar were evaluated 2, 4, 6, 8 and 10 weeks after inoculation (WAI). Each plant was examined in full to determine disease severity and the severity index was assigned to each plant based on the following standard scale of mosaic severity: plants without symptoms were assigned score “1”; plants with medium chlorotic spots or some distortion at the base were scored “2”; plants with spots on the whole leaf surface with leaf twisting were scored “3”; plants with distorted or shrunken leaf blades (to 2/3 of the leaf area) were scored “4”; and plants with many symptoms of CMD and/or total distortion of 4/5 of the leaf area and stunting of the entire plant were scored “5” [22].

### 2.5 Classification of cassava cultivars for resistance to CMD

Agglomerative hierarchical clustering was performed to classify cassava cultivars based on the disease severity scores at 10 WAI as described by Lokko et al. [23]. The severity score “1” was highly resistant (HR), severity score class [1.1–2] was resistant (R), severity score class [2.1–3] was susceptible (S) and severity score class [3.1–5] was highly susceptible (HS).

### 2.6 Virus and resistance gene identification in cassava cultivars

#### 2.6.1 DNA isolation

We collected young leaves from plants of the 24 cassava cultivars three months after inoculation. Leaves were packed in aluminum foil and placed in a cooler containing ice to limit degradation. The genomic DNA was extracted from fresh leaves, according to the protocol of Dellaporta et al. [24] with some modifications; 4 M ammonium acetate was used in the extraction and 2% polyvinylpyrrolidone was added to extraction buffer 2 (EB2). The fresh leaves were crushed with extraction buffer 1 (EB1) and EB2 in the mortars and then incubated for 1 h. DNA was precipitated in isopropanol alcohol for 2 h. The pellet of DNA resulting from the precipitation was rinsed twice with 700 μl of 80% ethanol and then dried at a temperature of 25–30 °C. The DNA was then dissolved in 50 μl of 1 × TE (Tris, EDTA) buffer and stored at −20 °C.

#### 2.6.2 Virus indexing by PCR

To determine the presence or absence of the cassava mosaic virus inoculated at each step of virus indexing, the sequence of coat protein of DNA-A was amplified from each virus in two cultivars of each category of resistance (HS, S, R and HR). The specific primers JSP1/JSP2 were used to amplify the coat protein sequence of all strains of ACMV, and JSP1/JSP3 were used to amplify the coat protein sequence all strains of EACMV [25]. The PCR cycling conditions consisted of an initial denaturation step of 94 °C for 5 min followed by 35 cycles of 45 s at 94 °C, 45 s at 55 °C, 55 s at 72 °C and a final step of 7 min at 72 °C. PCR was conducted in a total volume of 12.5 μl using 1.25 μl of MgCl_2_ (25 mM), 2.5 μl of 10 × PCR buffer (Qiagen, Hilden, Germany), 0.5 μl of each primer (10 μmol), 0.25 μl of dNTP (10 mM), 0.05 μl of Taq Polymerase (Qiagen HotStar Plus TM PCR) and 2 μl of DNA template (150 ng/μl). The PCR products were separated on 1.8% agarose gel using EZ-Vision (VWR International, Radnor, PA, USA) followed by UV revelation.

#### 2.6.3 Molecular resistance screening

The DNA of morphologically resistant cultivars was used for a second PCR using the marker pairs NS169 and RME1 associated with the *CMD2* resistance gene (Table 2). The 12.5 μl PCR volume contained 2 μl of DNA template (150 ng/μl), 0.25 μl of NS169/SSR and RME1/SCAR (5 μM), 0.25 μl of dNTPs (10 mM), 1.25 μl of 10 × PCR buffer, 1.25 μl of MgCl_2_ (25 mM) and 0.38 μl of Taq polymerase (Qiagen HotStar Plus TM PCR). The PCR was run under the following conditions: 2 min at 95 °C and 35 cycles of 30 s at 94 °C, 1 min at 55 °C and 1 min at 72 °C followed by a final elongation of 5 min at 72 °C for the marker NS169; and 2 min at 95 °C, 35 cycles of 30 s at 94 °C, 1 min at 50 °C and 1 min at 72 °C followed by a final elongation time of 5 min at 72 °C for the marker RME1. The PCR products were analyzed by electrophoresis in a 1.8% Agarose gel using EZ-Vision followed by UV exposure.

**Table 2:** Primers used for PCR detection of *African cassava mosaic virus* (ACMV) and *Eastern Africa cassava mosaic virus* (EACMV) and the molecular markers associated with resistance gene *CMD2.*

Type of virus and resistance genes	Primers	Marker system	Left primer 5′–3′	Right primer 5′–3′	Size of product (bp)	Annealing temperature (°C)
ACMV	JSP1/JSP2		ATGTCGAAGCGACCAGGAGAT	TGTTTATTAATTGCCAATACT	770	55
EACMV	JSP1/JSP3		ATGTCGAAGCGACCAGGAGAT	CCTTTATTAATTTGTCACTGC	770	55
*CMD2*	NS169	SSR	GTGCGAAATGGAAATCAATG	GCCTTCTCAGCATATGGAGC	319	55
	RME1	SCAR	ATGTTAATGTAATGAAAGAGC	AGAAGAGGGTAGGAGTTATGT	700	50

SSR, simple sequence repeat; SCAR, sequence-characterized amplified region.

### 2.7 Statistical analysis

A general linear model was used to correlate the number of successful grafts at 2 WAI and the disease severity score. The adjusted coefficient of determination (*R*^2^) was used to indicate the quality of the regression model. Analysis of variance was used to analyze the response of different cultivars to CMD, and Fisher's multiple comparison test at the 5% level was conducted to test for a significant difference among the disease severity scores of different cultivars. An agglomerative hierarchical clustering tree was generated using average disease severity to classify the 24 cultivars as described by Lokko et al. [23]. All analyses were performed in XLSTAT v.2014 (Addinsoft, Paris, France).

## 3 Results

### 3.1 Graft success rate

**Fig. 1:**
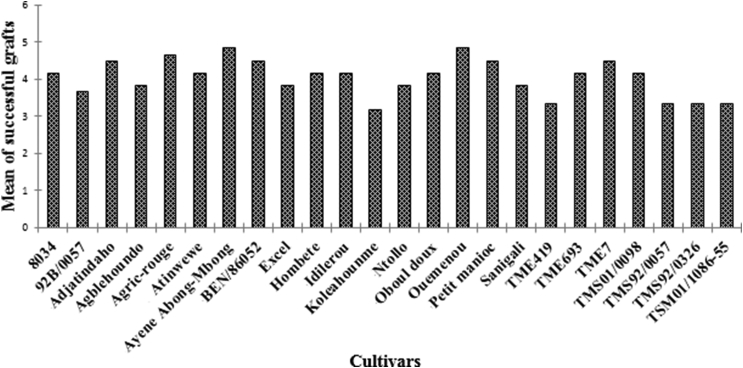
Mean number of successful grafts in each cassava cultivar (three plants with three graft replications per cultivar make a total of 216 plants grafted).

Fig. 1 shows the mean number of successful grafts of each cassava cultivar. The mean number of successful grafts at 2 WAI varied from 3.16 to 4.83 for each cultivar. The most successful grafts were observed in cultivars Ouemenou and Ayene Abong-Mbong (each with an average of 4.86 successful grafts). There was no significant difference (*P* = 0.22) between the successful grafting of different cassava cultivars at 2 WAI. Successful grafts were those that maintained the green color after two weeks. At least 65% of grafted buds were successful for different cultivars. No significant correlations (*P* ˃ 0.05) were found between the successful grafts and the mean disease severity of different cultivars (Table 3).

**Table 3:** Correlation coefficients between successful grafts and mean disease severity across all 24 cassava cultivars.

Variables	Grafts	2 WAI	4 WAI	6 WAI	8 WAI	10 WAI
Grafts	1					
2 WAI	0.10 (ns)	1				
4 WAI	0.07 (ns)	0.62*	1			
6 WAI	−0.06 (ns)	0.51*	0.74*	1		
8 WAI	−0.05 (ns)	0.41*	0.64*	0.85*	1	
10 WAI	−0.11 (ns)	0.15 (ns)	0.34*	0.58*	0.71*	1

ns, non-significant at *P* ≥ 0.05.

WAI, weeks after inoculation.

*, significant correlation (*P* < 0.05).

### 3.2 Severity of CMD

There was a significant difference between CMD severity scores of different cassava cultivars at 10 WAI (Table 4). At 2 WAI, a high mean severity was recorded in Ouemenou (2.17). Sixteen cultivars had a score of 1, two cultivars had a score of 1.50 and the other two cultivars had a score of 1.83. There was a significant difference between the mean CMD severity scores of the 24 cultivars (*P* < 0.0001). Eight cultivars (Hombete, 8034, Koleahonme, Ouemenou, TME 693, Idilerou, TMS92/0326 and Ayene Abong-Mbong) were the most susceptible to infection. At 4 WAI, 11 cultivars had a score of 1, six had a score greater than 1 and seven had a score greater than 2 (Table 4). The highest severity scores were in Ouemenou (mean of 2.67) and in Hombete, 8034 and Idilerou (mean of 2.50 for each).

**Table 4:** Severity of cassava mosaic disease (CMD) on 24 cultivars of cassava 2–10 weeks after inoculation (WAI).

	Mean CMD severity score				
Cultivar names	2 WAI	4 WAI	6 WAI	8 WAI	10 WAI
Hombete	1.83 ab	2.50 a	2.83 ab	3.10 a	3.10 a
8034	1.50 bc	2.50 a	3.08 a	3.16 a	3.20 a
Koleahonme	1.67 b	2.33 ab	2.83 ab	2.83 ab	2.67 abc
Ouemenou	2.17 a	2.67 a	2.83 ab	2.67 abc	2.50 abcd
TME 693	1.67 b	2.00 bc	2.33 cde	2.50 bcd	2.50 abcd
Idilerou	1.83 ab	2.50 a	2.66 abc	2.33 cde	2.17 cdef
Petit manioc	1.00 d	1.83 c	2.50 bcd	2.50 bcd	2.83 ab
Adjatidaho	1.00 d	1.83 c	2.33 cde	2.33 cde	2.17 cdef
TMS 92/0326	1.50 bc	2.00 bc	2.17 def	2.17 def	2.50 ef
Sanigali	1.00 d	1.33 d	2.00 efg	2.17 def	2.17 cdef
Ntollo	1.00 d	1.33 d	2.00 efg	2.00 ef	2.17 cdef
Excel	1.00 d	1.33 d	1.83 fgh	1.83 f	1.83 efg
Oboul doux	1.00 d	1.00 d	1.83 fgh	1.83 f	2.33 bcde
TME 419	1.00 d	1.00 d	1.67 ghi	2.00 ef	2.00 def
Agblehoundo	1.00 d	1.00 d	1.17 jk	2.00 ef	1.90 efg
TMS 92/0057	1.00 d	1.00 d	1.50 hij	1.83 f	2.00 def
Ayene Abong-Mbong	1.17 cd	1.17 d	1.33 ijk	1.33 g	1.67 fg
TMS 01/1086-55	1.00 d	1.00 d	1.83 fgh	1.83 f	1.83 efg
92B/0057	1.00 d	1.00 d	1.50 hij	1.83 f	2.00 def
BEN/86052	1.00 d	1.00 d	1.17 jk	1.83 f	1.83 efg
TMS01/0098	1.00 d	1.00 d	1.00 k	1.00 g	1.00 h
TME7	1.00 d	1.00 d	1.00 k	1.00 g	1.00 h
Atinwewe	1.00 d	1.00 d	1.00 k	1.00 g	1.00 h
Agric-rouge	1.00 d	1.00 d	1.00 k	1.00 g	1.00 h
LSD	0.43	0.48	0.47	0.46	0.52
*R*^2^	0.51	0.70	0.74	0.72	0.63
*F*	5.49	12.59	15.54	13.45	8.88
*P*	<0.0001	<0.0001	<0.0001	<0.0001	<0.0001

Different letters indicate significant differences between cultivars at *P* < 0.05 according to Fisher's multiple comparisons; *R*^2^ represents the coefficient of determination; and *F* represents the Fisher's values. LSD represents Fisher's least significant difference values associated with WAI.

At 6 WAI, mean severity scores also differed significantly among the 24 cassava cultivars (*P* < 0.0001). Four cultivars had a mean severity score of 1, nine had a mean severity score greater than 1, ten had a mean severity score greater than 2 and one had a mean severity score greater than 3 (Table 4). The highest mean disease severity (3.08) was observed in the cultivar 8034.

At 8 WAI, two cultivars (Hombete and 8034) had a mean severity score greater than 3 and 11 cultivars had a mean severity score greater than 2. Four cultivars had a mean severity score of 1 and six had mean severity scores greater than 1. A significant difference (*P* < 0.0001) was also observed between the mean severity scores of different cultivars. The cultivars Hombete and 8034 were the most susceptible to CMD infection with mean severity scores of 3.10 and 3.20, respectively (Table 4).

At 10 WAI, a significant difference of severity mean score (*P* < 0.0001) was observed under the different cassava cultivars. The highest mean severity scores of 3.10 and 3.20 were observed in Hombete and 8034, respectively.

### 3.3 Classification of cassava cultivars for resistance to CMD

**Fig. 2:**
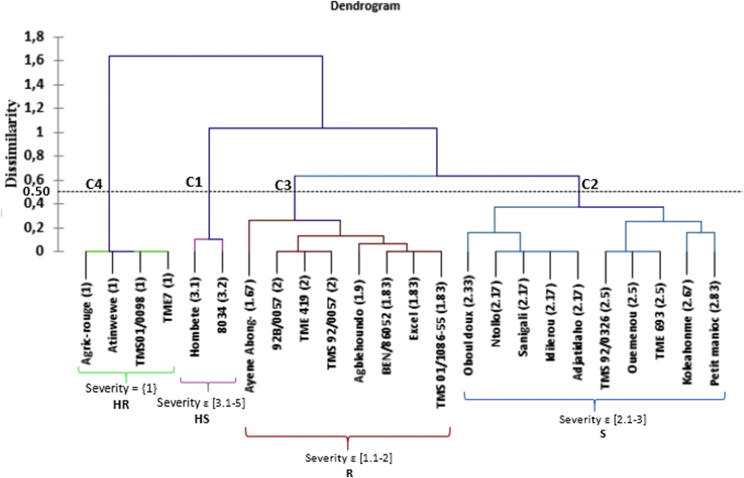
Cassava cultivar classification for resistance to CMD based on severity scores.

Based on disease severity scores, the 24 cultivars were classified into four groups: C1, C2, C3 and C4. Group C1 contained the cultivars with a mean severity score of 3.1–5 at 10 WAI; these cultivars were considered HS. Group C2 had mean severity scores of 2.1–3 and were considered S. Group C3 contained cultivars with severity mean scores of 1.1–2, considered R. Group C4 contained cultivars with severity score of 1, considered HR. A complete breakdown of cultivar classifications based on severity scores is provided in Fig. 2. In brief, the cultivars Adjatidaho, Agric-rouge, TME7 and TME01/0098 were HR; 92B/0057, Ayene Abong-Mbong, BEN86052, TMS92/0057, TMS419, Agblehoundo, Excel and TMS01/1086-55 were R; Hombete and 8034 were HS; and the remaining cultivars were S.

### 3.4 Virus detection in cassava cultivars

**Fig. 3:**
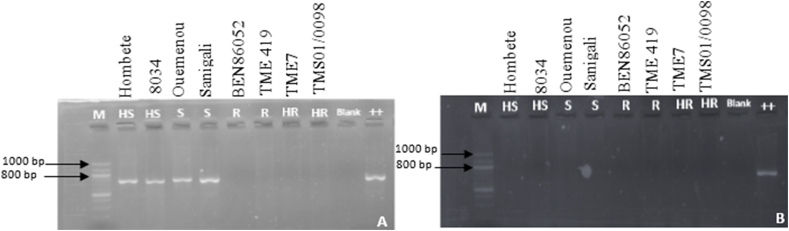
PCR amplification for virus indexing with the specific primers. (a) PCR amplification of *African cassava mosaic virus* (ACMV) coat protein with JSP1/JSP2 primers; (b) PCR amplification of *East African cassava mosaic virus* (EACMV) coat protein with JSP1/JSP3 primers using DNA from leaf samples of eight cultivars. The primers approximately amplified 770 bp of coat protein of each virus. HR = highly resistant, R = Resistant, S = Susceptible, HS = highly susceptible.

For ACMV, PCR analysis of leaf samples provided amplified products for the two HS cultivars and two of the S cultivars using the JSP1/JSP2 primers. PCR products were not observed in the two HR cultivars and the two R cultivars indexed (Fig. 3a).

The samples were also analyzed by PCR for eventual dual infestation by EACMV using the JSP1/JSP3 primers. No PCR products were observed in any of the eight cultivars (Fig. 3b); therefore, only ACMV was inoculated in this study.

### 3.5 Molecular screening

**Fig. 4:**
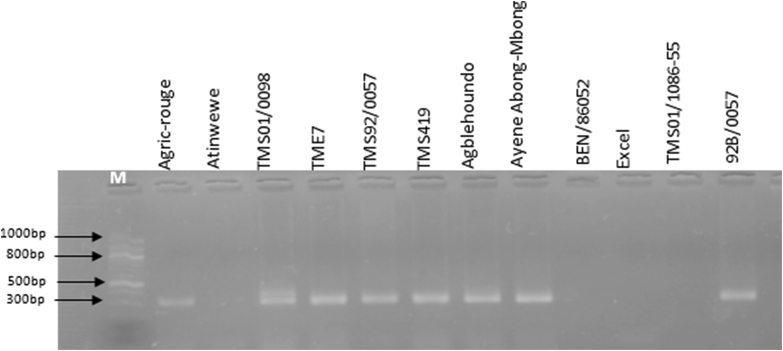
PCR detection of the molecular markers associated with the *CMD2* gene in highly resistant (HR) and resistant (R) cassava cultivars using the primer NS169 (SSR). The primers amplified 319 base pairs.

**Fig. 5:**
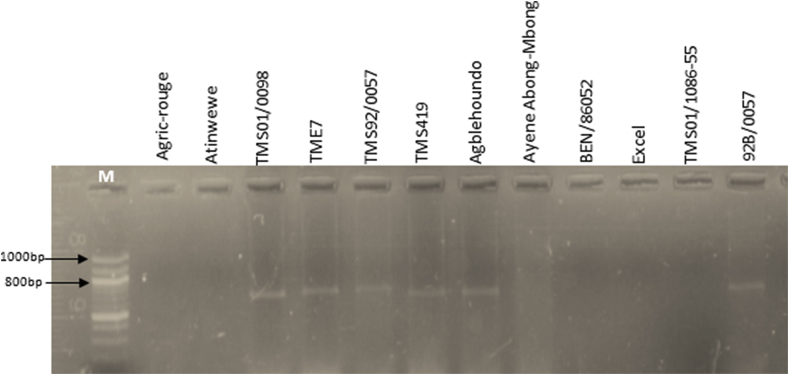
PCR detection of the molecular markers associated with the *CMD2* gene in highly resistant (HR) and resistant (R) cassava cultivars using the primer RME1 (SCAR). The primers amplified 700 base pairs.

Analyses using specific markers were conducted to confirm the resistance gene in plants. The molecular markers associated with *CMD2* were detected using the primer pair NS169 and RME1. The PCR products revealed the presence of *CMD2* in 8 of the 12 resistant cultivars (Agric-rouge, TMS01/0098, TME7, TMS92/0057, TMS419, Agblehoundo, Ayene Abong-Mbong and 92B/0057) using the primer NS169 (Fig. 4) and 6 of the 12 resistant cultivars (TMS01/0098, TME7, TMS92/0057, TMS419, Agblehoundo and 92B/0057) using the primer RME1 (Fig. 5).

In summary, the cultivars TMS01/0098 and TME7 were HR, indicating that the bands refer to the presence of the molecular markers associated with *CMD2* on the gels (Fig. 4, Fig. 5). The molecular markers associated with *CMD2* were revealed in the HR cultivar Agric-rouge only with NS169 and the HR cultivar Atinwewe completely lacked molecular markers associated with *CMD2* regardless of the primer used. The R cultivars TMS92/0057, TMS419, Agblehoundo, Ayene Abong-Mbong and 92B/0057 had lower CMD symptoms and showed evidence of containing *CMD2*, whereas BEN86052, Excel and TMS01/1086-55 showed lower CMD symptoms but did not appear to contain the *CMD2* gene whatever the primer (Fig. 4, Fig. 5).

## 4 Discussion

The high success rate of grafting (mean values of 3.16–4.83 among all cultivars) suggests that grafting is a good technique for transmitting viruses and for identifying virus-resistant cassava varieties [18,19,26]. However, the success rate of grafting did not statistically differ among cultivars and the success rate always exceeded 65%. These results were similar to those of Wagaba et al. [18], who also obtained a high success rate of grafting in a study of the efficiency of grafting in the transmission of viruses to cassava plants. This technique is therefore a powerful tool for rapid selection of cassava varieties against CMD.

There were significant differences in disease severity scores among cultivars. Cultivars that expressed the disease at 2 WAI were HS, which supports previous work showing that among susceptible cultivars, the first viral symptoms can occur within 2 WAI. In our study, symptoms appeared much earlier than for Wagaba et al. [18] who found the first symptoms 3–5 WAI. Earlier expression may be related to the high dose of the inoculum (six diseased buds for each graft) used in our study. Eight cultivars (Hombete, 8034, Koleahonme, Ouemenou, TME693, Idilerou, TMS92/0326 and Ayene Abong-Mbong) presented viral symptoms at 2 WAI; however, viral symptoms did not progress in Ayene Abong-Mbong. The severity of CMD increased in eight cultivars that showed symptoms at 2 WAI. Five cultivars showed symptoms by 4 WAI and seven cultivars showed symptoms by 6 WAI. It was previously shown that the time at which CMD symptoms manifest is not the same in all cassava cultivars [27].

The genetic background of cultivars is believed to be the key determinant in whether plants resist CMD. Thus, we sought a deeper understanding of the strains of cassava mosaic virus inoculated to each cultivar using molecular analyses. The primers JSP1/JSP2 and SJP1/JSP3 were used to amplify the coat protein of ACMV and EACMV, respectively. Only ACMV was identified in susceptible cultivars. It was previously reported that mixed infections of ACMV and EACMV cause severe disease symptoms [28,29]. A high disease severity can be caused by synergism or pseudo-recombination of several viruses within one plant [25,30]. The symptoms observed in the cultivars in this study may be associated with mixed infestations of several variants of ACMV, which could be detected by selecting specific primers in a follow-up study. For example, Asare et al. [16] detected 2–4 variants of ACMV in CMD-susceptible cultivars. In total, 12 cultivars were identified as resistant to CMD including the known R cultivars (TME7 and TMS01/0098). All these cultivars were further screened for resistance gene *CMD2* using their linked SSR and SCAR as markers [11,31,32].

Resistance genes to CMD have been reported in many studies [[33], [34], [35]] and used in cassava breeding programs to identify resistant cassava varieties [11,13,14,19,20]. Three resistance genes, *CMD1* (recessive), *CMD2* (dominant) and *CMD3* (QTL conferring resistance) [11,19,36] have been used in molecular CMD resistance screening of cassava, with *CMD2* being the most widely used [32,34]. Several markers have been linked to this resistance gene that facilitate breeding of CMD-resistant cultivars [20,23,31]. In this study, two markers NS169 and RME1 linked to *CMD2* [32,34,36] were used to confirm the presence of this resistance gene. We found that eight cultivars contained *CMD2*, including the known resistant cultivars TME7 and TMS01/0098. The surprising absence of bands related to the molecular markers associated with *CMD2* in the HR cultivars Atinwewe, BEN/86052, Excel and TMS01/1086052 suggests that the resistance of these particular cultivars is not caused by the presence of *CMD2*; instead, resistance to CMD may be conferred by other resistance genes in these cultivars [31,32,34,36,37]. Alternatively, the lack of *CMD2* in these HR cultivars could indicate that the primers used in this study were not reliable for *CMD2* detection in these particular cultivars [23]. It is possible that the CMD resistance in some cultivars is linked to other QTL that confer resistance, such as *CMD1* or *CMD3*, which were not studied here.

## 5 Conclusion

In this research, 24 cassava cultivars from Benin, Nigeria and Cameroon were screened for CMD resistance. Inoculation was performed by the grafting method and molecular markers linked to the *CMD2* resistance gene were used to detect potential mechanisms of resistance. Disease symptoms were highly expressed in susceptible cultivars and less expressed in some resistant cultivars. Of the 24 cassava cultivars screened against CMD, 12 cultivars were morphologically resistant. Further molecular screening of the 12 morphologically resistant cultivars showed that eight possessed *CMD2* and four did not. This work shows that *CMD2* is linked to the resistance of cassava cultivars and suggests it is worth searching for other QTL that confer resistance. The resistant cultivars identified in this work can help farmers to select resistant cultivars and ultimately better control CMD damage. These cultivars may also be prioritized for breeding programs to improve CMD resistance in other cassava cultivars.

## Author contributions

JAH, Z-TM, HBN, and SN designed the study; MJB and CA supervised the research; JAH, GHTC and SN conducted the work; JAH analyzed the data; JAH and MJB wrote the manuscript; JSP reviewed article; all authors read, corrected and approved the manuscript.

## Competing interests

The authors declare that they have no competing interest.

## Additional materials

All data generated or analyzed during this study are included in this published article.
